# Social influence on women’s contraceptive use: Population-based, sociocentric network study in rural Uganda

**DOI:** 10.1016/j.socscimed.2026.119145

**Published:** 2026-03-05

**Authors:** Alison B. Comfort, Sarah E. Piombo, James Moody, Charles Baguma, Bernard Kakuhikire, Esther Atukunda, Emily N. Satinsky, Mercy Juliet, E. Betty Namara, Phionah Ahereza, Cynthia C. Harper, Alexander C. Tsai

**Affiliations:** a Bixby Center for Global Reproductive Health, Department of Obstetrics & Gynecology, University of California San Francisco, 550 16th Street, San Francisco, CA, 94143, USA; b Department of Population and Public Health Sciences, Keck School of Medicine, University of Southern California, 1845 N Soto St, Los Angeles, CA, 90032, USA; c Department of Sociology, Duke University, 268 Soc/Psych Building, Durham, NC, 27708-0088, USA; d Mbarara University of Science and Technology, P. O. Box 1410, Mbarara, Uganda; e Department of Psychology, University of Southern California, 3620 McClintock Avenue, Los Angeles, CA, 90089, USA; f Center for Global Health and Mongan Institute, Massachusetts General Hospital, 125 Nashua Street, 7th floor, Boston, MA, 02114, USA; g Harvard Medical School, Boston, MA, Boston, MA, 02115, USA; h Department of Epidemiology, Harvard T.H. Chan School of Public Health, Kresge Building 9th floor, 677 Huntington Ave, Boston, MA, 02115, USA

**Keywords:** Contraception, Social influence, Social networks, Stochastic-actor oriented models, Peer interventions, Uganda

## Abstract

Myriad barriers impede women’s access to contraception in Uganda and affect their reproductive autonomy. Almost half of married women of reproductive age in Uganda want to avoid pregnancy and one-third are undecided, yet only 38% use modern contraception. Interventions harnessing the influence of women’s social networks may help to overcome these barriers and support desired contraceptive use. Using population-based longitudinal, socio-centric network data from household surveys across 8 villages in Uganda, we examined the role of social influence on contraceptive use among partnered, reproductive-age women. We fitted a stochastic actor-oriented model using three waves of data to estimate changes in social tie formation and contraceptive use over time as a function of contraceptive use among women’s social ties while adjusting for ego-level characteristics. We found that partnered women were 1.25 times more likely to choose contraceptive methods that were already popular in the network compared to methods that were less popular (p < 0.001). We identified a direct social influence effect on contraceptive use: women were 1.23 times more likely to choose a particular contraceptive method for each additional social tie who was using that same method (p < 0.05). Our findings fill a key evidence gap by being able to separate social ties’ tendencies to have similar contraceptive behaviors (i.e., homophily) in order to estimate the direct contribution of social influence on contraceptive use. These findings suggest that peer-based interventions focused on supporting contraceptive decision-making may be especially effective in overcoming barriers to contraception.

## 1. Introduction

Decision-making about contraception is influenced by a combination of personal, social, and societal factors. Social networks, meaning the social relationships individuals have with each other, can influence these decisions through multiple mechanisms. Interactions with others in one’s social network can lead to the exchange of knowledge, ideas, and opinions, which can influence behaviors (Valente, 2015). Social networks can also be conduits of social support and resources. Understanding the complex interpersonal dynamics surrounding family planning and contraceptive use is an important but under-researched area. Social influence arising through one’s social network may facilitate or impede contraceptive use, and understanding its role for contraceptive decision-making is important for ensuring that women can meet their reproductive needs.

Women from across African countries who want to avoid pregnancy continue facing barriers to family planning and contraceptive use. In Uganda, almost half of married reproductive-age women want to avoid pregnancy and a third are undecided, yet only 38% use modern contraception (Guttmacher Institute, 2017; Uganda Bureau of Statistics, 2023). Universal access to contraceptive services is a key priority of the 2030 Sustainable Development Goals (United Nations, 2020). Ensuring women can meet their reproductive health care needs will allow them to exercise their reproductive autonomy, choosing if and when to have children (Ross and Solinger, 2017), and will reduce the persistently high rates of pregnancy-related deaths in Africa (Guttmacher Institute, 2017).

The barriers to contraceptive use specifically in African contexts span several domains, including individual, inter-personal, and structural barriers to use. At the personal level, factors including age, level of education, socio-economic status, rurality, and religious beliefs are important, based on systematic reviews of quantitative and qualitative studies from across African countries (Blackstone et al., 2017; Engelbert Bain et al., 2021). Lack of correct knowledge about contraceptive methods, especially misperceptions about these methods and their side effects, also limit contraceptive use from data across many different African settings (Blackstone et al., 2017; Campbell et al., 2006; Diamond-Smith et al., 2012; Engelbert Bain et al., 2021). This systematic review citing several examples from African contexts confirmed that one of the main reasons for not using contraceptive methods is due to fears of future infertility, which is especially relevant in these settings where fertility is highly valued (Blackstone et al., 2017). For example, some women associate intra-uterine devices (IUD) and hormonal methods with infertility, leading to hesitancy in using them (Blackstone et al., 2017). One recently published study in Uganda found that women were reluctant to use hormonal methods because they believed it would negatively impact future fertility (Kawuma et al., 2023). Concerns about contraceptive methods’ effects on future fertility are often based on actual health and side-effects. Most reversible methods of contraceptives have an immediate return to fertility, except for injectables, which are long-acting hormonal medications that have a delay in return to fertility. Recent evidence confirmed that injectables had the longest delays for return to fertility after stopping use, while IUDs and implants had the shortest delays; but across all methods, there were no lasting effects on future fertility (Yland et al., 2020). Yet misperceptions of permanent effects on future fertility are often cited as reasons for not using them.

Inter-personal barriers include women’s lack of autonomy with decision-making within the household, domestic violence and reproductive coercion, couple discordance about fertility intentions and contraceptive preferences, and social norms within the community regarding contraceptive use according to these systematic reviews and other studies from several African countries (Blackstone et al., 2017; Challa et al., 2018; Engelbert Bain et al., 2021; Hung et al., 2012; Stephenson et al., 2007). Structural barriers to contraceptive use highlighted in these studies of African countries include: lack of access to confidential, high quality reproductive and sexual health care, exposure to family planning education and information campaigns, transportation barriers to access, gender-inequitable norms, and availability and affordability of contraceptive methods and services (Challa et al., 2018; Engelbert Bain et al., 2021; Stephenson et al., 2007).

While many studies have examined personal and structural factors related to use, fewer have quantitatively examined the role of social influence through inter-personal factors. Understanding the relationship between social networks and reproductive health outcomes is important as many of the barriers to contraceptive decision-making – at the individual, inter-personal and structural level – may be affected by women’s social networks. Yet there remains an important knowledge gap in establishing temporality while accounting for potential selection effects, due to the tendency for women to form social ties with other people who are similar on observable and unobservable characteristics. Most studies are cross-sectional and unable to clarify temporal ordering of exposures and outcomes (see Prior empirical support), while those using longitudinal approaches are dated and provide examples from only two country contexts, Kenya and Bolivia (Behrman et al., 2002; Valente and Saba, 2001). The existing literature makes clear that social networks may operate differently depending on social contexts, so there is a need for context-specific evidence that uncovers the mechanisms through which social networks affect contraceptive use.

No studies have tested for social influence effects on contraceptive use while adjusting for individual characteristics, type of contraceptive method, and changes in network structure over time. To address this gap, we collected longitudinal, socio-centric network data from the 8 villages of a rural parish in Uganda. Using longitudinal data on contraceptive use among partnered, reproductive-aged women from three survey waves and combining these data with complete network data from all adults in the study villages, we estimated a stochastic actor-oriented model (SAOM). This rigorous longitudinal network analytic approach allows us to simultaneously account for (a) the effects of network structure, including the tendency to have social ties to others who are similar, (b) personal attributes, and (c) social ties’ influences on contraceptive use. This approach specifically allows us to estimate the temporal effect from having social ties who use contraceptive methods during one time period and estimating the effect on a woman’s use of that contraceptive method in the following period, a temporal effect which is not possible in previously published cross-sectional studies of social networks and contraceptive outcomes.

Ultimately, establishing the role of social network influence on contraceptive use, and understanding the underlying mechanisms, will draw attention to the need to engage these social networks in supporting women’s contraceptive decision-making.

## 2. Conceptual framework

Following the Social-Ecological Model (Bronfenbrenner, 1977; Dahlberg and Krug, 2002) and frameworks proposed by Holt et al. (2024), Challa et al. (2018), Costenbader et al. (2019) and Shakya et al. (2021) for contraceptive decision-making, factors at the individual-, inter-personal, community, and policy level can contribute to contraceptive decision-making (Bronfenbrenner, 1977; Challa et al., 2018; Costenbader et al., 2019; Dahlberg and Krug, 2002; Holt et al., 2024; Shakya et al., 2021). At the individual-level, women vary in their fertility preferences and pregnancy intentions, knowledge, attitudes, and beliefs. Women also differ in contraceptive method preferences (e.g. privacy of use, concerns about side effects, effectiveness) and related factors that affect contraceptive decisions (e.g., age, education level, socio-economic status, religious affiliation).

At the inter-personal level, social relationships play an important role, particularly relationships to others with experience using different methods. These relationships allow women to learn from their peers’ experiences with contraception, reduce uncertainty about different methods, provide support for (or pressure against) using contraception, and inform women about personal network norms (Bongaarts and Watkins, 1996). Partner-related factors, including quality of partner relationship, female autonomy and decision-making within the household, and couple discordance about fertility preferences, also affect contraceptive decision-making.

At the community-level, social norms around fertility, sexual activity, and contraceptive behaviors contribute to contraceptive decision-making, as do gender, religious, and cultural norms related to family planning (Challa et al., 2018; Stephenson et al., 2007). Finally, within the health, economic, political and socio-cultural context, factors such as community sensitization about family planning, distance to health care facility, and availability of confidential, high quality sexual and reproductive health services (including access to full range of methods) play a role for contraceptive decision-making (Challa et al., 2018; Stephenson et al., 2007).

Our study specifically focuses on the role of social networks on contraceptive decision-making. Social network theory posits that the social relationships individuals have with others, including the structure and composition of these networks, can affect attitudes, beliefs, and behaviors in complex ways, beyond the effects of individual-level attributes (Berkman et al., 2000; Granovetter, 1973; Moreno, 1934; Valente, 2010). Social network analysis is a defined theoretical perspective and methodology used to analyze and understand the changes in these relationships and the influence that they can have on individual- and group-level outcomes (Valente, 2010). In social network analysis, the focal individual is referred to as the *ego*, and the network members they have social ties with are referred to as *alters*.

**Fig. 1.**
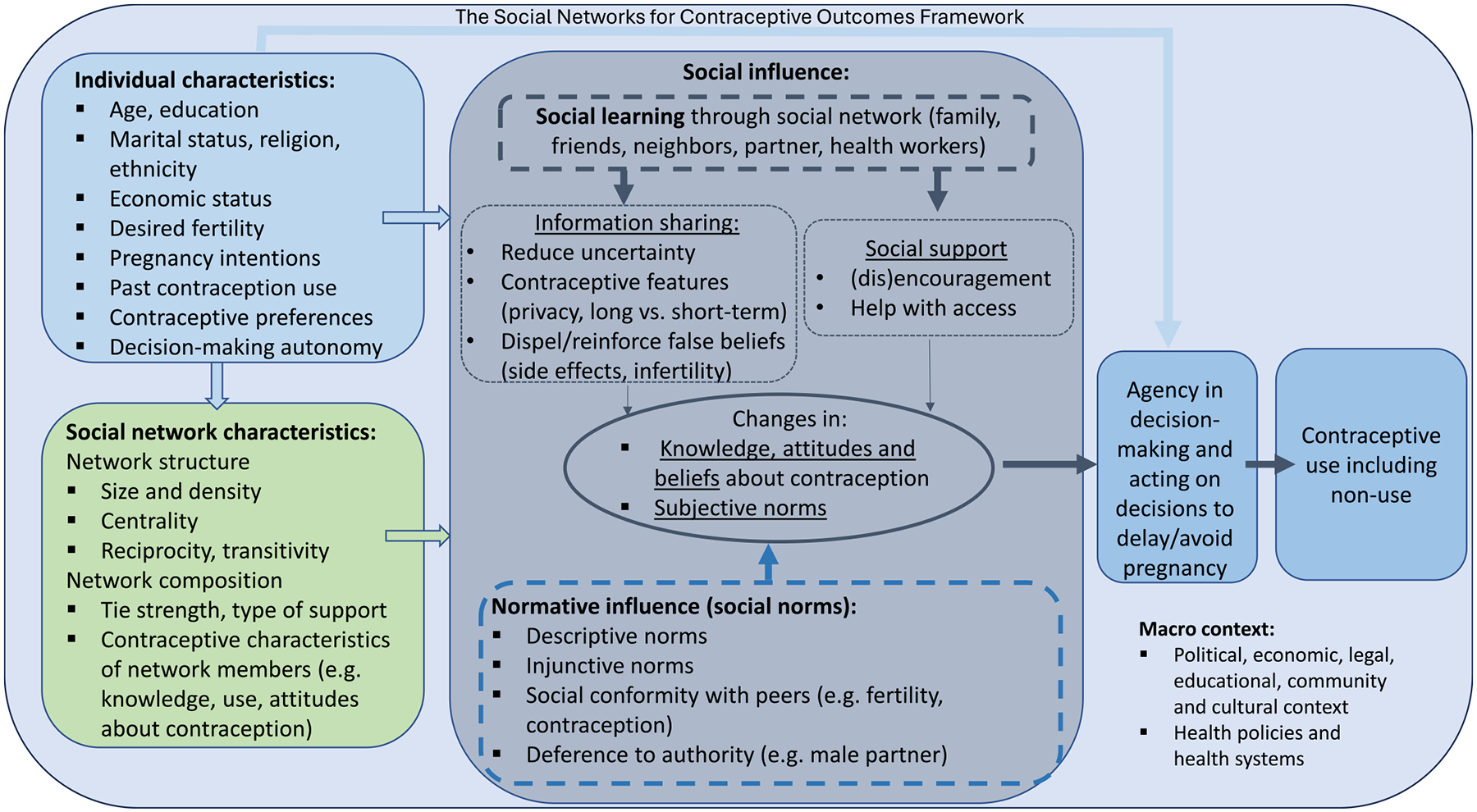
The social networks for contraceptive outcomes framework.

We present a conceptual framework, The Social Networks for Contraceptive Outcomes Framework (Fig. 1),that relates social networks to contraceptive decision-making, considering and extending the model developed by Berkman et al. (2000) on networks and health, the model by Montgomery and Casterline (1996) on social influence and fertility, and the newly proposed Contraceptive Agency Framework by Holt et al. (2024). Social networks themselves vary in structure (e.g., size, density, centrality) and composition (e.g. type of social support from alters, tie strength etc.). In our conceptual framework, composition will also depend on alter attributes, for example the percentage of others in network using contraception or encouraging/discouraging contraceptive use. The structure and composition of these networks exist within and may be affected by the broader macro context. They will also be affected by individual attributes such as sociability, fertility preferences, marital status and other characteristics. In turn, social networks, through their structure and composition, can affect contraceptive-related attitudes, beliefs, and behaviors through several mechanisms. This conceptual model is intended to complement and expand the existing models whereby social influence via social network composition and structure affects both agency in decision-making related to avoiding or delaying pregnancy and agency in acting on those decisions.

### 2.1. Hypothesized social network mechanisms of action

Our hypothesized mechanisms of action are depicted in Fig. 1 - The Social Networks for Contraceptive Outcomes Framework. Social networks can operate through *social influence,* following theories of social learning and diffusion of innovations (Bandura, 1986; Rogers, 2003). Social influence occurs when individuals’ attitudes, beliefs, and behaviors change as a result of interactions with others in their social network. Here, we describe three social influence mechanisms through which networks could affect contraceptive decision making: information-sharing (*social learning),* normative influence of peers (*social norms),* and social support. Typically, peer modeling is also included as a mechanism for social influence. However, we left out this mechanism because contraceptive use is not often a visible behavior to be emulated, as in the case of overt behaviors like smoking.

*Social learning* can occur both through close social ties and through observation of more distant social ties. Social network members can encourage or discourage the use of contraception. They can also share information about availability of different contraceptives, how to use them, potential benefits and side effects, effectiveness, other contraceptive features, and anecdotal experiences (Montgomery and Casterline, 1996). They may not necessarily share correct information about contraceptive methods, as social networks can also be sources of misinformation related to reproductive health behaviors (Comfort et al., 2022). Network theory suggests that contraceptive adoption, including the types of methods being used, depends on the prevalence of contraceptive use within a network and the types of methods being used, which will vary across settings (Kohler, 1997).

Another channel through which social networks can influence contraceptive decision-making is through the provision of *social* support, which can include emotional support, financial and instrumental support (e.g., providing transport to access clinics), informational support (e.g., social ties providing advice), and appraisal support (e.g., evaluating different possible decisions) (Cohen and Wills, 1985). The beneficial effect of social support can either be indirect (i.e., serving as a buffer against stressors (Cassel, 1976; Cobb, 1976) or direct.

Beyond these channels of influence of individual social network members, the composition of the network (e.g., whether others in a network are using contraceptive methods and what types of methods are being used) and its structure (e.g. density) may contribute to social influence related to contraceptive decision-making (Granovetter, 1973; Valente, 1995). Network density refers to how connected network members are and is calculated as the ratio of actual connections out of all possible connections in a network (Valente, 2010). Sparse networks are networks with low density, or fewer ties present. Network density can impact the flow of information and behavior change in networks. For example, sparse networks will have more independent information sources and are likely to exert less pressure for conformity to normative behaviors, whereas dense networks may provide redundant information while also creating greater pressure to conform to normative behaviors (Kohler et al., 2001). Social learning may be especially important in contexts where there is not widespread knowledge about or access to a broad range of contraceptive methods, and in contexts where there may be mistrust in the health system. However, identifying the contribution of social learning to contraceptive decision-making is challenged by the potentially confounding tendency for individuals to associate with others who are similar on both observable and unobservable characteristics (i.e., homophily (McPherson et al., 2001)) as well as the common exposure to similar environmental influences (i.e., shared environment). In this study, the parish social network is a shared environment. Contraceptive method choice within the parish is shaped by some of these shared environment factors, such as access to the same contraceptive methods and distance from healthcare facilities. Specifically in Uganda, the health care system is structured as a tiered system where the Health Center II provides outpatient care including contraceptive methods, specifically oral contraceptive pills and injectables, female and male condoms and fertility awareness methods; these methods are also available through the local drug shops in the villages, while the Health Center III provides access to implants and IUDs (Ssanyu et al., 2025).

*Social norms* represent another primary domain of social influence. These include both injunctive norms (such as perceptions of others’ approval of contraceptive use) and descriptive norms (perceptions of the prevalence of contraceptive use within a community) (Cialdini et al., 1991). Social norms around fertility and contraception can influence contraceptive decision-making through the perceptions of what others within a social group or community believe or do, in line with the Theory of Planned Behavior (Ajzen, 1991; Montgomery and Casterline, 1996). For example, individuals may tend to follow the attitudes and behaviors of those in their social group to feel a sense of belonging (Rimal and Real, 2003). Depending on the social norms around fertility and contraceptive use in the community, women may either receive support to act on their fertility- and contraceptive-related desires or they may be pressured or even prevented from acting on these desires (Montgomery and Casterline, 1996).

### 2.2. Prior empirical support for the conceptual framework

Most studies examining the role of social networks in contraceptive use have identified a positive association between contraceptive attitudes and behaviors within a woman’s social network and her own contraceptive use (Behrman et al., 2002; Comfort et al., 2021a; Dynes et al., 2012; Gayen and Raeside, 2010; Islam, 2019; Kincaid, 2000; Kohler et al., 2001; Madhavan and Adams, 2003; Musalia, 2005; Shakya et al., 2020; Valente et al., 1997). Studies have also shown that, while health providers play an important role for contraceptive education and provision, individuals often prefer to rely on their social networks for contraceptive information and advice (Boulay and Valente, 1999; Comfort et al., 2021a,b; Mosha and Ruben, 2013). Several studies have explored the role of social influence on contraceptive use. These studies have found that both social and network compositional factors are associated with contraceptive outcomes. Indeed, a systematic review of studies on social networks and reproductive health in low-resource countries identified 18 observational studies, most of which were cross-sectional with limited ability to establish a causal relationship between social networks and reproductive outcomes (Lowe and Moore, 2014). While contraceptive use among social ties has been shown to be associated with women’s own contraceptive use, it is sometimes the perceptions of contraceptive use among social ties that is more predictive of contraceptive use, even if this perception is incorrect (Valente et al., 1997). Studies have also highlighted social ties’ support for contraceptive use as being more predictive of an individual’s contraceptive use than the social ties’ *actual* use (Shakya et al., 2020; Valente et al., 1997). The composition of these social networks (i.e., whether they include those supportive of contraceptive use or not) can explain these findings, with differences identifying according to whether the network includes certain social ties (e.g., conjugal kin versus individuals from outside the village) (Madhavan and Adams, 2003; Musalia, 2005).

While these studies have explored the role of social influence on contraceptive outcomes, they are primarily cross-sectional and do not explore contraceptive use within the context of the evolution social networks. Most of these studies are unable to rule out reverse causality whereby women who use contraception may be more likely to form social ties with others who support and use contraception. One of the few studies that used longitudinal data and adjusted for potential homophily in tie formation did find that social influence through social learning still played a role in explaining contraceptive outcomes, after accounting for selection effects (Behrman et al., 2002).

Several studies have also explored the role of social norms in contraceptive decision-making. Evidence from several different African country contexts has shown that injunctive norms around family planning and contraception, including community attitudes around fertility and contraceptive use, play a role in contraceptive decision-making (Challa et al., 2018; Costenbader et al., 2019; Dynes et al., 2012). Descriptive norms also play an important part in predicting both intentions to use contraception and actual contraceptive use (Costenbader et al., 2019; Kohler et al., 2001). Social norms can contribute to either supporting or preventing women from acting on their reproductive goals, depending on whether social norms around contraceptive use differ from a woman’s own intentions. Kohler et al. (2001) explored whether social learning or social norms explained contraceptive use across different communities and found that, in certain locations, social learning dominated while in other contexts social norms were more predictive of contraceptive use. In those contexts, social norms either facilitated or impeded contraceptive use, depending on whether contraceptive use was the normative behavior in that location (Kohler et al., 2001).

Finally, fewer studies have explored network structure and its contribution to explaining contraceptive use. Centrality is an important measure of one’s position in a network. Central individuals are highly connected within the network and often have greater access to information and influence on others. In contrast, peripheral individuals have fewer connections and may have less influence and information access. Network theory suggests that more individuals who occupy more central positions in their networks may be earlier adopters of a new innovation (e.g., a newly introduced contraceptive method) because they are well integrated in their communities and may therefore have access to more information (Coleman et al., 1966; Rogers and Kincaid, 1981). However, individuals on the periphery of their networks (i.e., and who are less central) may be less constrained in adopting behaviors that are not common in the network (e.g., using contraception or adopting a new contraceptive method) (Becker, 1970). One of the earliest studies on family planning indeed found that central individuals were earlier adopters of contraceptive methods (Rogers and Kincaid, 1981). A more recent study exploring network structure found that more central individuals were more likely to use contraception and that their contraceptive use was associated with contraceptive use among others in the network. This study was also among the few that conducted analyses by method type; it was found that women tended to have social ties to others using similar contraceptive methods (Gayen and Raeside, 2010).

### 2.3. Stochastic actor-oriented models and contraceptive decision-making

This conceptual and empirical review demonstrates clear interest in understanding the extent to which social influence can explain contraceptive decision-making. Yet there is limited recent evidence capable of establishing this relationship. The stochastic actor-oriented model (SAOM) offers several advantages over traditional regression models and offers a promising and heretofore unused approach to empirical inquiry into social influence and contraceptive use. SAOMs were designed to statistically estimate changes in social networks over time from the perspective of the individuals or “actors” (Ripley et al., 2023; Snijders et al., 2010). These models are agent-based simulations applied to longitudinal data, which predict changes in tie formation between each wave of data based on the state of the network at that time point, an individual’s own characteristics, and the characteristics of others in the network. SAOMs also allow us to model the co-evolution of multiple networks, meaning that we can apply them to simultaneously predict both social tie formation and behaviors over time. These models can quantify changes in social ties based on individual or network factors and quantify changes in behavior based on individual and social factors. This approach has been used to understand the role of social influence in a range of health outcomes and health behaviors including physical activity (de la Haye et al., 2011), tobacco use (de la Haye et al., 2019; Green et al., 2013; Haas and Schaefer, 2014; Lakon et al., 2015; Schaefer et al., 2012), e-cigarette use (Piombo et al., 2025), alcohol use and delinquency (McMillan et al., 2018), marijuana use (de la Haye et al., 2015), malaria prevention (Vörös et al., 2025), and sexually transmitted infections (Young and Fujimoto, 2021). They have not yet been applied to contraceptive decision-making. To address this gap in the literature, we used SAOMs to model changes in social networks and contraceptive use among partnered women to understand the impact of social ties on contraceptive use.

## 3. Methods

### 3.1. Study site and study participants

Our study was conducted in rural southwestern Uganda, where the population primarily relies on subsistence agriculture, animal husbandry and small-scale trading for income-generation (Takada et al., 2019). Given that the informal sector makes up a large part of economic activity, social networks – including kinship ties, friendship ties, and ties to neighbors and community members – are especially important for information sharing, offering social support (emotional, information, instrumental, and appraisal) and mutual assistance, enforcing community norms and creating opportunities for social engagement. In Uganda, individuals tend to belong to different types of community-based groups providing mutual aid and support such as burial groups, savings groups and other community associations (Musinguzi et al., 2022; Peterlechner, 2009). These different forms of social capital have been shown to be important for poverty reduction in Uganda (Hassan and Birungi, 2011). In our specific study context, individuals are part of vocational groups (e.g. beekeeping and basket-weaving groups), savings groups, church-based groups, informal gardening groups, local councils and parish development groups, and special interest groups (e.g. positive living groups for individuals living with HIV) (Kakuhikire et al., 2021).

We conducted a socio-centric, longitudinal study in Nyakabare Parish, a rural region in southwestern Uganda (Takada et al., 2019). Starting in 2014, data were collected every 2 years for a total of three survey waves. All adults in the 8 study villages (individuals aged 18 years or older or emancipated minors aged 16–18 years) who considered Nyakabare Parish their primary residence were eligible, with minimal exclusion criteria (e.g. cognitive impairment, acute intoxication). Emancipated minors were individuals ages 16–18 who were either cohabitating with a partner, had a child, were pregnant, or were responsible for their own livelihood. At each survey wave, study participants were added (age eligibility, in-migration) or removed (deceased, out-migration). Eighty-five participants who were missing data on village location and demographic characteristics were removed, yielding an analytical sample of 2217 individuals across the three survey waves. Data collectors visited the study participants at their homes to collect data using a tablet-based survey. They first obtained written informed consent. Almost all study participants consented to participate (91.6% in wave 1, 90.8% in wave 2, and 89.5% in wave 3). The survey was conducted in the study participants’ local language (Runyankole). Study participants received either 1 kg of sugar or a bar of soap for study participation, consistent with local norms for conducting research in this area. The study received approval from Massachusetts General Hospital IRB (study #2013P000395), Mbarara University of Science and Technology IRB (study #07/10–12), with research reliance agreements from the University of California San Francisco and University of Southern California.

Residents of Nyakabare Parish have access to the local health clinic, known as a Health Center II, where contraceptive services are available. Methods available at this health center include oral contraceptive pills and injectables, male and female condoms and fertility-awareness methods. The implants and IUDs are available at the Kinoni Health Center IV, which is ~9.5 km away. Because our sample included all contiguous villages in the parish, study participants would have similar access to contraceptive methods at the Health Center II and Health Center IV.

### 3.2. Data collection

Our analysis is restricted to the first three waves of data collection when contraceptive use data were collected from partnered women of reproductive age. These women included those who were either married (legally or through traditional ceremony) or currently living with their partner. They were asked whether they or their partner had used any contraceptive method in the past 12 months to prevent pregnancy or transmission of a sexually transmitted infection. For those responding yes, they were asked to name which methods. Options included male and female condoms, oral contraception, injectables, intrauterine device (IUD), male or female sterilization, withdrawal, or the rhythm method. Due to an administrative error, the implant was omitted as a stand-alone item in the list of options. The research assistants collecting the data marked those who reported using implants as using injectables (i.e. because both methods are inserted into the arm). Due to this error, our results combine injectables and implants, which are the first and second most popular methods in Uganda (Uganda Bureau of Statistics, 2023) into a single contraceptive category. Data from the full sample of study participants also included socio-demographic and economic characteristics, including age, sex, formal education, religion, self-reported HIV status, desired number of children, parity, household asset wealth (Smith et al., 2020), frequency of family planning discussions with partner (“more than twice per year” vs. less frequently or not at all), quality of partner relationship and decision-making in the household (if applicable), and village fixed effects. While the contraceptive use outcome was only measured among partnered women, our analytical sample included all adults in the study to account for formation of ties with any other study participants and the influence of alter attributes on tie formation.

### 3.3. Measures

#### 3.3.1. Social network

At each wave of data collection, we collected social network data using five culturally-adapted name generators covering health, leisure, money, emotional support, and food exchange (Takada et al., 2019). Study participants could nominate up to six adults residing in the parish in each name generator. For example, the name generator used to solicit alters in their health network was the following: “Over the past 12 months, with whom in this parish have you usually discussed any kind of health issue? Examples of health issues might include topics like your child’s health, family planning, nutrition, HIV, mental health, immunizations, sanitation methods, alcohol abuse or other issues.” We constructed a directed network at the three time points, inclusive of all villages, using the nominations from all name generators. A nomination from one individual to another was represented by a 1 (tie present) or a 0 (absent).

#### 3.3.2. Contraceptive method network

**Fig. 2.**
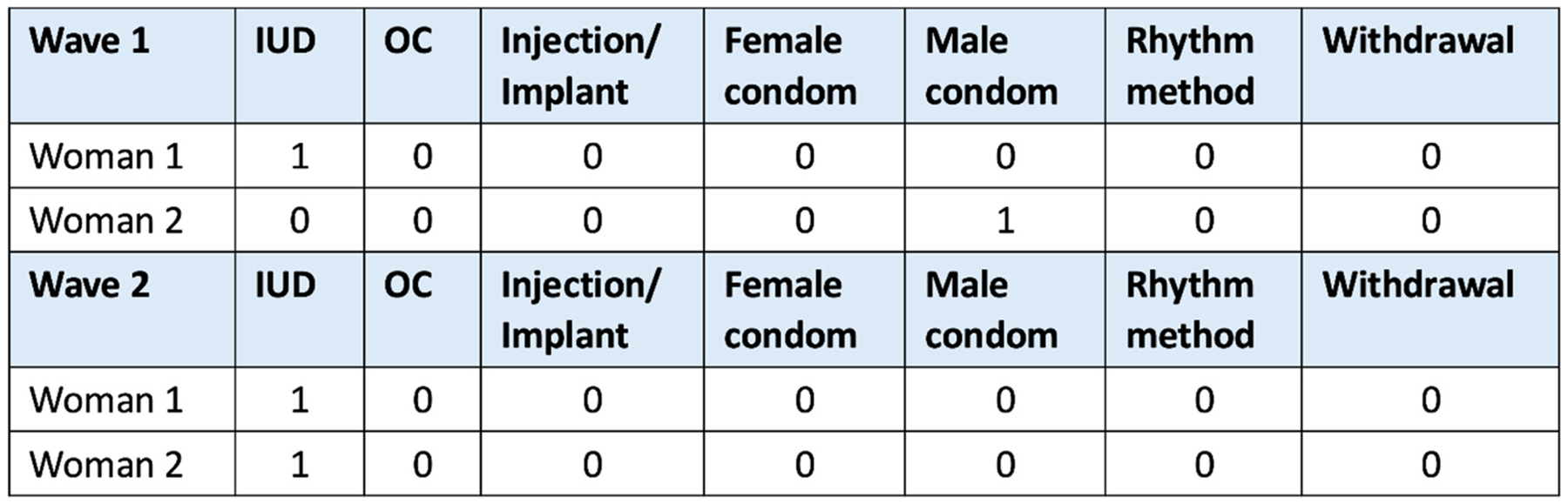
Example matrix of woman-method network. OC= oral contraceptive; IUD= intra-uterine device

We conceptualized women’s contraceptive method use as an affiliation network, or two-mode network, with partnered, reproductive-age women (first mode) choosing among all possible contraceptive methods (second mode) including the option of selecting none. This approach follows Fujimoto et al. (2018) and Schaefer et al. (2022) on the co-evolution of one-mode and two-mode networks. We implemented a SAOM in the Simulation Investigation for Empirical Network Analysis software package (RSiena version 1.3.14–17) (Ripley et al., 2023; Snijders et al., 2010) to examine changes in tie formation and contraceptive method use based on local network structures, individual attributes, and social influence processes. The primary outcome of interest was contraception use, equal to 1 for each method used and 0 otherwise (e.g., IUD = 0/1, pill = 0/1 etc.). The data are represented as matrices, with rows representing the individuals and columns representing contraceptive methods (see Fig. 2 for example). Changes in contraceptive behavior are represented as changes in woman-method ties in the two-mode network across waves. Structural zeros were used in the model to indicate that a tie was not allowed for certain individuals. For the contraceptive method network, structural zeros were included for men, non-partnered women, and women over the age of 49 (because these subgroups of participants in the parent study were not administered the survey module about contraceptive use). Therefore, these individuals have no bearing on the contraceptive network parameter estimates.

#### 3.3.3. Contraceptive method variables

Each contraceptive method was assigned to the following categorical attribute types: IUD, female and male sterilization, injectables and implants, oral contraceptives, barrier methods (male and female condoms), other methods (rhythm method and withdrawal), or no contraceptive method. Each method was represented independently in the two-mode network, but the attribute type was used to test hypotheses about certain methods being more or less popular among women. These attributes can be thought of as covariates for the contraceptive methods (e.g., if IUDs were more popular relative to injectables and implants, oral contraceptives, or barrier methods).

### 3.4. Analytical approach

Using a SAOM implemented in RSiena (Ripley et al., 2023; Snijders et al., 2010), we modeled the co-evolution of both the Nyakabare Parish social network and partnered women’s contraceptive use over time. This approach uses an agent-based simulation algorithm to simultaneously model changes in the parish social network (the one mode network consisting only of social ties between individuals) and changes in the contraceptive method network (the two-mode network, consisting of “ties” between partnered women and their contraceptive method choices) (Snijders et al., 2013). The agent-based simulations calculate changes in the network occurring between each wave through a series of ministeps. At each ministep, actors can make changes in their social network (forming or dissolving social ties). In the contraceptive network, actors can make changes at each ministep by deciding whether to continue using a certain method, stop using it, or switch contraceptive methods. The algorithm simulates changes in social tie formation and contraceptive method use for individuals based on individual attributes, influence from others in the network, and local network structures. Score-tests were used in forward model selection to determine inclusion of relevant “effects” (or components of the objective function used to model probabilities of change in the network). The final model met convergence criteria, with an overall maximum convergence ratio <0.25. All individual model parameter t-ratios were <0.10, consistent with standards for SAOM (Ripley et al., 2023; Snijders et al., 2010).

#### 3.4.1. Specification of the social network

Several network and individual level effects were included based on model requirements and research aims. This portion of the model estimates changes in social tie formation in the greater network to provide insight into factors contributing to tie selection, such as homophily effects or the tendency to form ties with others who are similar to oneself. The social network contained effects for outdegree (representing the balance between the formation and dissolution of social ties), reciprocity (representing how often ties are reciprocated), geometrically-weighted edgewise shared partnerships (GWESP; representing transitivity, or the tendency to form ties with the social ties of others in one’s network), indegree popularity square root (the tendency of popular individuals to continue receiving nominations), outdegree popularity (the tendency of actors with high outdegree to receive more nominations), and outdegree activity (the tendency of actors with high outdegree to continue sending more nominations). Individual level attributes were included as three separate effects: sender (“covariate-ego”, the tendency for individuals with this attribute to send more nominations), receiver (“covariate-alter”, the tendency to receive nominations), and homophily (“covariate-same”, the tendency to form ties based on attribute similarity). Sender, receiver, and homophily effects were included for individual covariates, including sex, education, religion, HIV status, and asset index. A homophily effect was included for living in the same village, as was an effect for similarity on age.

#### 3.4.2. Specification of the contraceptive method network

The two-mode network contained endogenous network effects, individual covariate effects, and social influence effects. The endogenous network effects included the outdegree effect representing the overall probability of using a contraceptive method and the indegree popularity effect representing the tendency for more women to use contraceptive methods that are already popular in the network. The effects of individual attributes were included to evaluate the effects on contraceptive use of education, religion, HIV status, household asset wealth, and frequent family planning discussions with the woman’s partner. An effect for the tendency to choose IUDs over other method types was also included in the model. A direct influence effect representing social network influence on contraceptive method use was included. This effect has a temporal component, measuring whether women who were connected in the social network at the previous time point were more likely to choose the same contraceptive method as their social ties at the next time point. For example, in wave 2, if a woman was not using oral contraceptives and was friends with an alter using oral contraceptives, the social network influence effect would estimate the likelihood of that woman using oral contraceptives by wave 3 (i.e. selecting the same method as her alters). When a woman is considering choosing a contraceptive method, the direct influence effect increases with the number of alters who currently use that method in the prior wave. For someone not using a method who has one alter using oral contraceptives and one alter using an IUD, there is equal direct influence for both methods. Any difference in predicted choice is driven by other covariates. If IUD has greater indegree popularity (i.e. more women in the network using that method) than oral contraceptives, then the IUD would be the predicted method of choice, with all other covariates being held equal.

The model also had an interaction term between use of IUDs and social influence to test if direct influence was occurring differentially for IUDs. Inclusion of this interaction term was motivated by the fact that IUDs were the method becoming more popular over time, therefore we felt it was important to include the interaction effect to test whether the social influence effect was only being driven by this type of contraceptive method.

## 4. Results

### 4.1. Summary characteristics of study participants

In the population cohort, there were a total of 1819 study participants enrolled at baseline, with an additional 398 enrolled in subsequent waves. In the cohort, most (55%) were women, fifty-nine percent were either married or cohabitating with a partner, and the average age was 38 years old. At baseline, there were 450 married or cohabiting women under the age of 50 years (Table 1). At subsequent waves, additional women joined the study, and others left the study or were outside reproductive age at follow up, resulting in 426 women in the 24-month survey and 407 in the 48-month survey. The average age for the women at baseline was 32 years old with an average of 4.2 children. Most (55%) completed at least a primary education. The prevalence of HIV was 9%. Most study participants identified as Catholic or Protestant (79%).

**Fig. 3.**
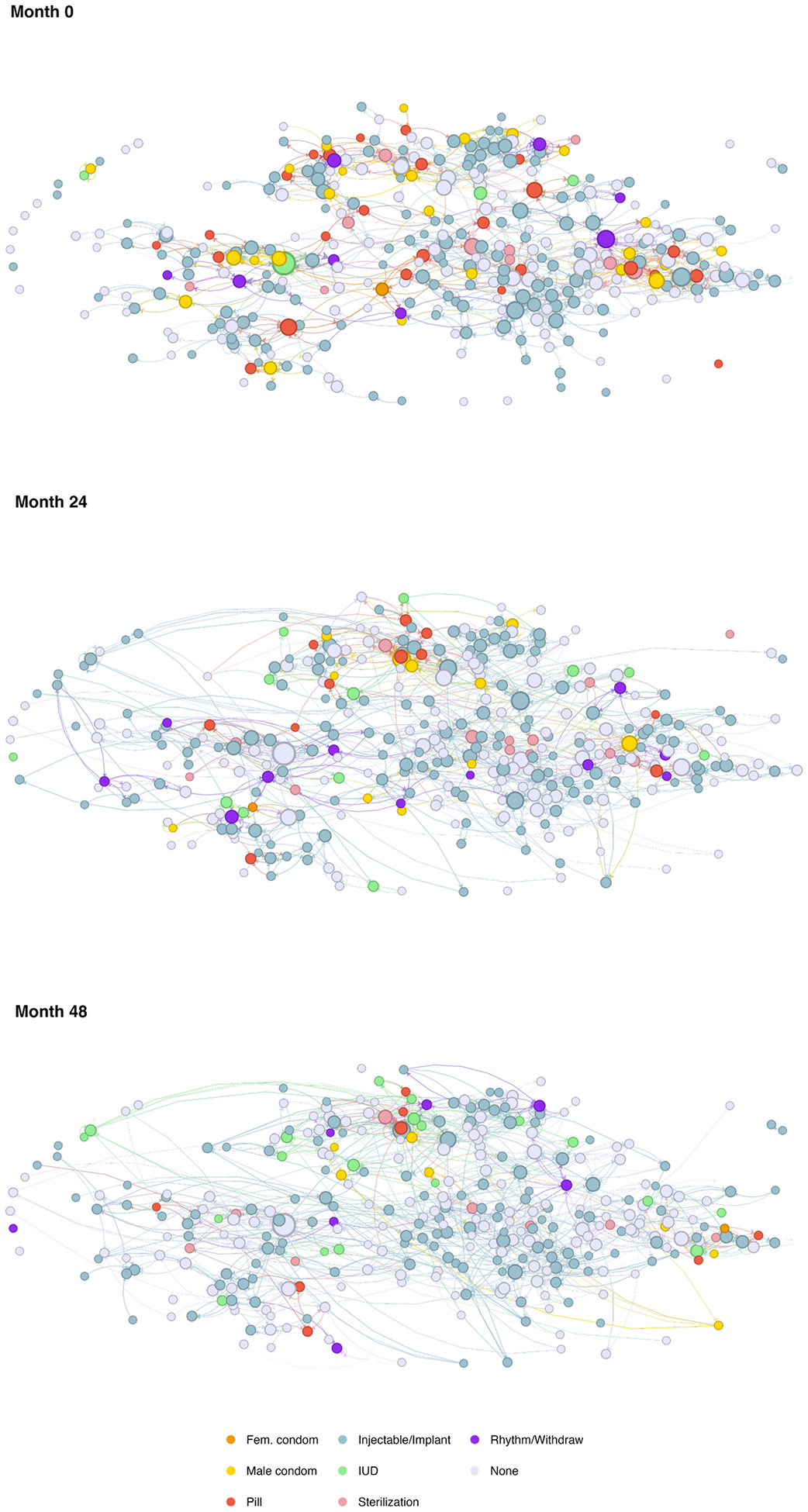
Social Networks and Contraceptive Use Among Partnered, Reproductive Age Women at Each Wave. Notes: Nodes are sized proportionate to their number of social ties and colored by contraceptive method.

Most of the women (59%) reported contraception use in the last year. The proportion of women using contraception did not change over time (61% at wave 2 [24-months post-baseline] and 59% at wave 3 [48-months post-baseline]) (Table 2). At baseline, the most popular contraceptive methods were primarily injectables and implants (45%) followed by oral contraceptives (6%) (Fig. 3). By the third wave, there was a trend toward increased use of IUDs from 2% in the first wave to 7% in the third wave. There was also a trend toward decreased use of oral contraceptives (from 6% at baseline to 2% in the third wave). On average, women in the sample had 4.2 children [IQR 2–6]). Among partnered individuals, 55% (26% of sample) said they discussed family planning often with their partner.

### 4.2. Parish social network

The SAOM showed statistically significant effects for network dynamics and individual covariates (Table 3). The negative outdegree parameter indicated that there was lower density or greater network sparseness. Since density is a measure of actual ties out of all possible ties, for a large network it is typical to have lower density (i.e., in a network of thousands of people, it is unlikely that you will have social ties to many of them). The positive reciprocity and GWESP effects indicated that ties were more likely to be reciprocated and that there was a tendency to form ties with the social ties of others in one’s network. The positive indegree popularity effect meant that popular individuals in the network continued receiving nominations and became more popular over time. In other words, these individuals’ popularity is self-reinforcing as they continue to form more social ties over time. The negative outdegree popularity effect showed that individuals were less likely to attract or maintain more social ties over time as a result of sending many nominations.

There was evidence of homophily in tie formation based on sex, education level, religion, HIV status, age, and asset index: study participants were more likely to form social ties with others who were more similar to themselves based on these attributes, compared to having ties to others who were different on these attributes. For example, women were 1.79 (exp[0.58]) times more likely to form social ties with other women rather than men (adjusted odds ratio [aOR] 1.79). People with HIV were also more likely to form ties with each other rather than with others of different serostatus. We also found evidence of homophily by village and by age, meaning that individuals were more likely to form ties with others in the same village or other of similar age rather than with those in the other study villages or others of different ages.

Women were less likely to send social tie nominations compared to men (Table 3 female-ego). Individuals with greater household asset wealth were less likely to send social tie nominations compared to less wealthy individuals (AIndex-ego) and individuals were more likely to have social ties with others who have the same household asset wealth (AIndex same). Additionally, individuals living with HIV were more likely to both send and receive social tie nominations (HIV status ego, HIV status alter).

### 4.3. Contraceptive method network

**Fig. 4.**
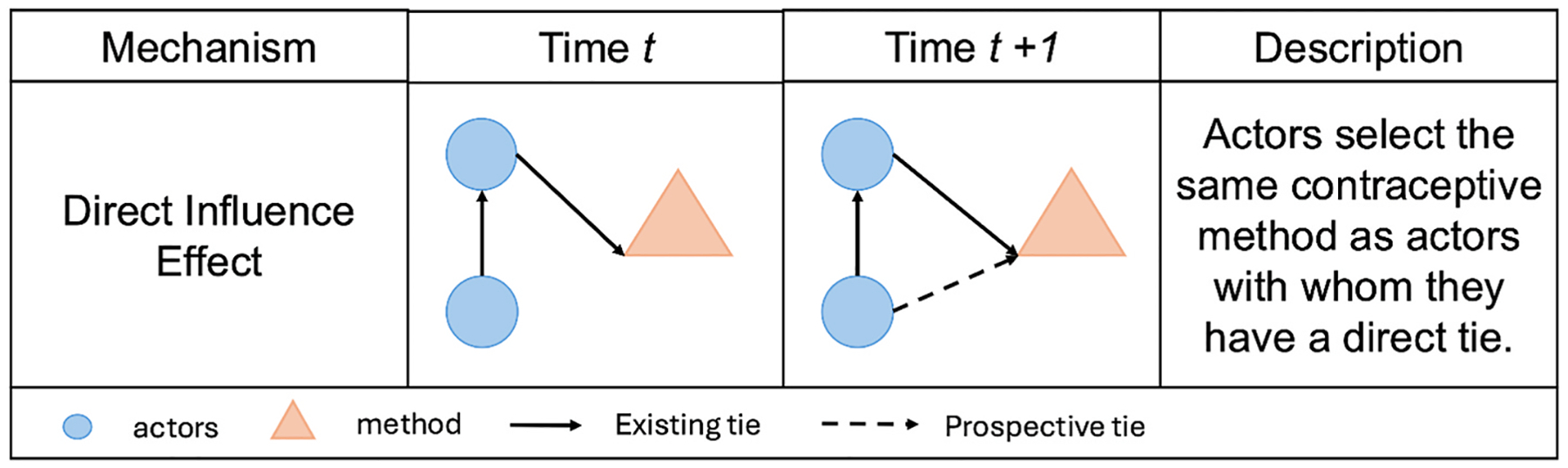
Direct influence effect diagram. Adapted from Schaefer et al., (2022).

In the contraceptive method network, the positive indegree popularity effect showed that for each one unit increase in the square-root of a method’s indegree (i.e., number of women using that method), partnered women had a 1.25 (exp[0.22]) times higher odds of selecting that method, holding other effects constant. For example, if the number of women using a given method increases from 9 women to 16 women (square root of 9 = 3, square root of 16 = 4) then this would reflect a one unit increase in indegree popularity and correspond to a 25% increase in the odds of selecting that method. This indicates that contraceptive method options with higher uptake among women are more likely to attract additional users over time. This effect is reflective of overall network trends in the popularity of different contraceptive method types. The model also identified a significant direct influence effect on contraceptive use. Women were more likely to select a contraceptive method if other women to whom they are socially connected (their alters) were already using that option. Partnered women were 1.23 (exp [0.21]) times more likely to choose a particular contraceptive method for each additional social tie who used that method, after adjusting for ego characteristics (Fig. 4. This direct influence effect indicates that women tend to adopt contraceptive methods used by their alters, net of overall method popularity. Taken together, these effects demonstrate both broad and local network influence.

Given the steady increase in IUD use over time (Table 2), we estimated an effect to specifically examine IUD use (vs. other methods) and an interaction term between the IUD and direct influence to determine if social influence was differential for the IUD compared to all other methods. This interaction was significant, indicating that social influence effects were stronger for IUD use compared to other methods. Each additional alter using an IUD was associated with 2.97 (exp[1.09]) times higher odds of IUD selection among women, net of overall method popularity and other covariates. Together, these findings indicate there was a statistically significant social influence effect across contraceptive method types, but this effect was stronger for IUD use.

## 5. Discussion

In this population-based cohort study of partnered women in rural Uganda, we find evidence of social influence effects on contraceptive use. After adjusting for network structural and ego-level characteristics, we identify that partnered women are more likely to choose the same contraceptive method if other women in their social network also used the same method. By fitting a SAOM to several waves of data, we are able to separate out homophily in contraceptive behaviors to estimate the contribution of social influence on contraceptive behaviors. By modeling contraceptive method use as a two-mode network, we can show not only the contribution of social influence to contraceptive use, but also demonstrate that women are aligning their contraceptive method choice with others in their social network. Our model also accounts for the tendencies for popular contraceptive methods (e.g., IUDs) to increase in popularity compared with less popular methods (e.g., male condoms), while adjusting for individual-level factors that are typically predictive of contraceptive use.

Our approach substantially improves on other studies examining the role of social networks in contraceptive behaviors by demonstrating the contribution of social networks within a rigorous longitudinal framework. This model provides evidence that partnered women are more likely to select the same contraceptive method as other partnered women in their network, highlighting the importance of social networks for sharing information, advice, and support on reproductive health decision making (Comfort et al., 2021a;^2022^). A strength of the approach is that the modeled social influence effect provides stronger evidence than a binary outcome because there is evidence that women are not only choosing whether or not to use contraceptives but also choosing to use the same method as their social ties. Most studies examining the role of social networks on contraceptive use fit multivariable regression models to cross-sectional data (Comfort et al., 2021a; Dynes et al., 2012; Gayen and Raeside, 2010; Islam, 2019; Kincaid, 2000; Kohler et al., 2001; Madhavan and Adams, 2003; Musalia, 2005; Shakya et al., 2020; Valente et al., 1997). Few studies have used longitudinal data to examine social influence (Behrman et al., 2002; Valente and Saba, 2001). Albeit dated, the study by Behrman et al. (2002) used longitudinal data from both women and men in Kenya and included individual fixed effects to account for potential unobserved preferences to form social ties with similar individuals and to account for potential correlation in unobserved characteristics among social ties. They found that social influence through social learning persisted even after accounting for selection effects (Behrman et al., 2002). However, traditional regression models do not necessarily account for dependencies in network data. In comparison, the SAOMs used in the current study are designed specifically for longitudinal network analysis. Our model brings forth the tendency within this study population to form homophilous ties along several characteristics, while also moving beyond cross-sectional associations to demonstrate more conclusively the sequential contribution of social influences on contraceptive method use.

There are several different pathways through which the observed social influence could occur, including through information-sharing among peers, social norms, and other forms of social support. While our data do not permit us to separate which mechanism is contributing to these social influence effects, other studies examining social influence show that different mechanisms could be occurring. One study from Cameroon found that perceptions of alters’ contraceptive use were, and support from alters, more predictive of contraceptive use than alters’ actual contraceptive use (Valente et al., 1997). Several studies from other country contexts (Kenya, Niger, and Mali) have confirmed that support (or lack of support) among alters was an important factor predicting contraceptive use (Madhavan and Adams, 2003; Musalia, 2005; Shakya et al., 2020). It is important to highlight that the contribution of social influence may, in certain contexts, conflict with the fertility and contraceptive-related intentions of women. For example, one study from Kenya found that when women’s social networks were composed of non-family members, contraceptive use was lower because those members did not support contraceptive use. Our data do not permit us to identify which mechanism is contributing to these social influence effects. We do include partner-related factors, which have been shown to play a role in contraceptive decision-making (Nkonde et al., 2023), by adjusting for frequency of partner discussions about family planning, but the effect was not statistically significant. Nonetheless, our findings highlight the importance of identifying ways in which to engage social networks so that they are more supportive of women’s fertility and contraceptive intentions.

Few studies have examined specific contraceptive methods in relation to social influence. A study in Bangladesh found that more central individuals within the network tended to use contraception and that the method they used was similar to that of their alters (Gayen and Raeside, 2010). A model proposed by Kohler (1997) on contraceptive adoption suggests that the types of contraceptive methods that will be used within a given context are conditional on whether and what type of contraceptive methods are already being used within the network, since these will be the methods that women discuss and share information about (Kohler, 1997). Our results are consistent with this hypothesis because we find that women tend to choose contraceptive methods already popular within the network (e.g. injectables), which suggests s certain level of information and advice-sharing about these particular methods.

Our findings from the full parish network, inclusive of men and non-partnered women, also confirm the tendency for people to form social ties with others similar to them (e.g., on characteristics such as age, sex, religion, serostatus, economic status, and village of residence). These tendencies provide insight into network dynamics that affect social tie formation. Modeling the co-evolution of the greater network provides context for the social environment that women making contraceptive decisions are embedded in. The homophily findings in the overall network demonstrate that individuals are more likely to associate with others similar to themselves on multiple characteristics, which may be an important factor to consider in network-based intervention design. Overall, our findings provide some of the first longitudinal evidence of social influence on contraceptive use, where contraceptive method use by the social ties of partnered women preceded the same contraceptive method selections of these partnered women. These results mean that peer-based interventions to support contraceptive decision-making may be especially promising for reducing barriers women face in meeting their reproductive needs and intentions.

This study advances our understanding of the contribution of social influence on contraceptive use. However, we acknowledge the limitation of our outcome variable (use of contraceptive methods). Studies exploring the role of social networks on contraceptive outcomes have also focused on contraceptive use as the outcome of interest (Behrman et al., 2002; Comfort et al., 2021a; Dynes et al., 2012; Gayen and Raeside, 2010; Islam, 2019; Kohler et al., 2001; Madhavan and Adams, 2003; Mosha and Ruben, 2013; Musalia, 2005; Shakya et al., 2020; Valente et al., 1997). The field of reproductive health acknowledges that this outcome measure does not adequately capture contraceptive preferences and reproductive autonomy (Burke and Potter, 2023; Holt et al., 2023, 2024; Senderowicz, 2020). Assessing contraceptive use does not convey whether the method being used is the *desired* contraceptive method, or whether the individual wants to use contraception at all (Burke and Potter, 2023). Women using a particular method may desire to use a different method but face barriers to switching; some women using a method may prefer not to use any method while others who are not using may want to start using (Holt et al., 2023; Rocca et al., 2024). Finally, the measure does not demonstrate the extent to which women have autonomy in enacting their decisions related to contraception (Harper et al., 2023; Holt et al., 2024). Our data were collected as early as 2012 through 2016 and we did not benefit from these important changes in the field of reproductive health to better conceptualize contraceptive decision-making. Future research exploring the role of social influence on contraceptive decision-making could greatly benefit from integrating these new measures.

Our study has several additional limitations. First, the findings reflect partnered women because the outcome was collected among this sub-sample of women. Understanding the role of social influence for contraceptive decision-making among unpartnered women is important and warrants further research. Second, our study was restricted to individuals 18 years or older, or emancipated minors, and findings do not reflect the role of social influence among adolescents who are becoming (or who already may be) sexually active. This population is important to study, especially given their higher risk of pregnancy. Third, as discussed, the outcome measure has significant limitations in reflecting contraceptive decision-making and women’s reproductive autonomy. We included an effect for discussion of family planning with partner to capture women’s communication with their partner about these topics. Future analyses would benefit from additional data being collected to reflect measures of preference-aligned contraceptive outcomes (Holt et al., 2025), Desire to Avoid Pregnancy Scale (Rocca et al., 2019) and decision-making dynamics related to contraceptive autonomy (Senderowicz, 2020). Fourth, our analyses distinguish between contraceptive method types, yet (as discussed above in Methods) implants were grouped together with injectables due to an administrative error in separating these two responses. Research assistants confirmed that, while the names of each method differ in the local language (Runyankole), if a study participant reported using an implant, the research assistants recorded it as injectable since they are both placed in the arm. This grouping of methods means that we cannot distinguish between social influence effects relative to these two methods. Together, these two methods are the most popular methods used by Ugandan women (Uganda Bureau of Statistics, 2023). Future research distinguishing between the two is important. Fifth, while modeling contraceptive method use as a two-mode network provides greater insight about method selection compared to modeling a binary outcome, there are certain limitations. Our analysis is not able to account for social influence effects coming from unpartnered women in the study population, since contraceptive data were not collected from them. However, the model does include unpartnered women as well as men to look at their contribution to social tie formation in the greater parish network. Lastly, our conceptual framework provides a comprehensive view of the pathways through which social networks contribute to contraceptive outcomes. Our analyses take advantage of the unique opportunity to implement a SAOM using longitudinal data with complete (sociocentric) network data to disentangle the contribution of social influence on contraceptive use. However, this approach means that we are not able to assess the contribution of other pathways including injunctive norms, nor were we able to adjust for individual attributes such as pregnancy intentions which were not included in the data.

## 6. Conclusions

Social influence within networks plays an important role in explaining contraceptive use. Women are learning from others in their social networks about different contraceptive methods and adopting the methods used by others in their network. Information-sharing, advice, support, and norms around contraceptive use within these social networks play an important role for contraceptive outcomes. These findings suggest that it is critical to engage these social networks in interventions that aim to ensure women can meet their reproductive needs. At the same time, it is important to consider how social networks may impede women from meeting their fertility- and contraceptive-related intentions, especially in contexts where social norms around fertility and contraception may differ from women’s own desires. The broader social network, especially other women such as friends, mothers, mothers-inlaw, and sisters, may also have an important role in contributing information, advice, and support for women’s contraceptive decisions. Engaging social networks is a promising way to help women reach their reproductive intentions, desires, and outcomes.

## Figures and Tables

**Table 1:** Demographic and behavior characteristics of reproductive age women at baseline (N = 450).

	N (%) or Mean (SD)
Age	32.1 (8.2)
Number of children	4.2 (2.5)
Education	
None or some primary	204 (45.3)
Completed primary/primary +	246 (54.7)
Asset Index (Baseline)	
1 (least affluent)	98 (21.8)
2	104 (23.1)
3	92 (20.4)
4	88 (19.6)
5 (most affluent)	65 (14.4)
Missing	3 (0.7)
Religion	
Christian (Protestant or Catholic)	358 (79.6)
Muslim	7 (1.6)
Non-religious/other	27 (6.0)
Missing	58 (12.9)
Discuss family planning with partner	
More than twice per year	277 (61.6)
Never/once or twice	164 (36.4)
Not applicable	0 (0)
Missing	9 (2.0)
Self-reported HIV positive	42 (9.3)

**Table 2:** Contraceptive method use in past year among partnered reproductive age women^a^.

	Wave 1 (N %)	Wave 2 (N %)	Wave 3 (N %)
Modern contraceptive use	59.3	61.3	58.5
Contraceptive pill	29 (6.4)	18 (4.2)	9 (2.1)
Injection/Implant	202 (44.9)	204 (47.9)	186 (45.7)
IUD	8 (1.8)	25 (5.9)	27 (6.6)
Male or female sterilization	12 (2.7)	17 (4.0)	9 (2.2)
Male condom	26 (5.8)	11 (2.6)	8 (2.0)
Female condom	1 (0.2)	1 (0.2)	1 (0.3)
Rhythm method	2 (0.4)	6 (1.4)	3 (0.7)
Withdrawal	14 (3.1)	10 (2.4)	6 (1.5)
No contraceptive method	169 (37.6)	148 (34.7)	155 (38.1)

a Total sample size varies by wave, see Table 1.

**Table 3:** Estimates from stochastic actor-oriented model for the co-evolution of social network and contraceptive method network (N = 2217).

Effect	Parish Social Network	Contraceptive Method Network
	Estimate (SE)	Estimate (SE)
*Essential Effects and Structural Controls*		
Rate (period 1)	16.57 (0.45)	2.79 (0.26)
Rate (period 2)	11.78 (0.32)	2.29 (0.21)
Outdegree (density)	−4.27 (0.09)***	−3.18 (0.14)***
Reciprocity	2.16 (0.07)***	
GWESP	1.25 (0.03)***	
Indegree - popularity (sqrt)	0.30 (0.06)***	0.22 (0.01)***
Outdegree - popularity (sqrt)	−0.54 (0.07)***	
Outdegree - activity (sqrt)	−0.11 (0.01)***	
*Individual attributes*		
Female alter	−0.02 (0.02)	
Female ego	−0.15 (0.02)***	
Female same	0.58 (0.02)***	
Education alter	−0.03 (0.02)†	
Education ego	−0.02 (0.02)	0.02 (0.12)
Education Same	0.08 (0.02)***	
Religion alter	−0.17 (0.04)***	
Religion ego	−0.19 (0.04)***	0.00 (0.18)
Religion same	0.43 (0.04)***	
HIV status alter	0.19 (0.03)***	
HIV status ego	0.12 (0.03)***	0.07 (0.18)
HIV status same	0.26 (0.03)***	
Asset index alter	0.02 (0.01)†	
Asset index ego	−0.03 (0.01)***	0.02 (0.04)
Asset index same	0.15 (0.02)***	
Same Village	1.37 (0.02)***	
Age similarity	1.63 (0.07)***	
Discuss family planning with partner		0.02 (0.12)
Number of children ego		0.02 (0.02)
*Contraceptive Method Attributes*		
IUD		0.25 (0.23)
*Between Network Effects*		
Direct influence (social network to contraceptive method agreement)		0.21 (0.09)*
Direct influence X IUD		1.09 (0.52)*

† p < 0.1;

* p < 0.05;

** p < 0.01;

*** p < 0.001.

All convergence t ratios <0.09. Overall maximum convergence ratio 0.18.

## Data Availability

The authors do not have permission to share data.
